# Safety and feasibility of tumor treating fields initiated before and during radiotherapy for newly diagnosed glioblastoma: results from the Arm A feasibility cohort of a phase I/II trial (PriCoTTF)

**DOI:** 10.1186/s12885-026-16613-y

**Published:** 2026-07-27

**Authors:** Sied Kebir, Christoph Oster, Sandeep Sharma, Leon Jekel, Teresa Schmidt, Jana Grieger, Giorgio Cappello, Kathrin Kizina, Lazaros Lazaridis, Laurèl Rauschenbach, Yahya Ahmadipour, Philipp Dammann, Andreas Junker, Kathy Keyvani, Jonas Feldheim, Daniela Pierscianek, Björn Scheffler, Martin Proescholdt, Peter Hau, Anca-Ligia Grosu, Dietmar Krex, Ulrich Sure, Christoph Kleinschnitz, Christoph Pöttgen, Martin Stuschke, Martin Glas

**Affiliations:** 1 Department of Neurology and Center for Translational Neuro- and Behavioral Sciences (C-TNBS), Division of Clinical Neuro-Oncology, University Medicine Essen, University of Duisburg-Essen, Essen, Germany; 2 Department of Neurosurgery and Spine Surgery, University Medicine Essen, University of Duisburg-Essen, Essen, Germany; 3 Department of Neurosurgery, University Hospital Regensburg, Regensburg, Germany; 4 Department of Neurology and Wilhelm Sander Neuro-Oncology Unit, University Hospital Regensburg, Regensburg, Germany; 5 Department of Radiation Oncology, Medical Center - University of Freiburg, Freiburg, Germany; 6 Department of Neurosurgery, University Hospital Carl Gustav Carus, Technical University Dresden, Dresden, Germany; 7 DKFZ Division of Translational Neurooncology at the West German Cancer Center (WTZ), German Cancer Consortium (DKTK) Partner Site, University Medicine Essen, Essen, Germany; 8 German Cancer Research Center (DKFZ), Heidelberg, Germany; 9 Department of Neurology and Center for Translational Neuro- and Behavioral Sciences (C-TNBS), University Medicine Essen, University of Duisburg-Essen, Essen, Germany; 10 Department of Radiotherapy, University Medicine Essen, University of Duisburg-Essen, Essen, Germany; 11 Department of Neurology, University Hospital Knappschaftskrankenhaus Bochum, Ruhr University Bochum, Bochum, Germany; 12 Institute of Neuropathology, University Medicine Essen, University Duisburg-Essen, Essen, Germany; 13 Department of Neurology and Neurooncology, St. Marien Hospital Lünen, Lünen, Germany; 14 Department of Neurosurgery, St. Marien Hospital Lünen, Lünen, Germany; 15 Department of Neurology, University Hospital Nuremberg, Paracelsus Medical University, Nuremberg, Germany

**Keywords:** Glioblastoma, Tumor Treating Fields, TTFields, Radiotherapy, Safety, Feasibility, Phase I/II trial

## Abstract

**Background:**

Tumor Treating Fields improve survival when started after chemoradiotherapy in newly diagnosed glioblastoma, but the safety and feasibility of initiating treatment before and during radiotherapy remain uncertain.

**Methods:**

PriCoTTF is a prospective, open-label, nonrandomized, multicenter phase I/II trial. Arm A enrolled adults aged 18 to 70 years with newly diagnosed IDH-wildtype glioblastoma or gliosarcoma, Karnofsky Performance Status of at least 60, and planned focal radiotherapy to 60 Gy in 30 fractions. TTFields at 200 kHz were initiated 2 to 4 weeks after surgery and 1 to 2 weeks before radiotherapy. The primary endpoint was protocol-defined treatment-limiting toxicity from TTFields initiation through 4 weeks after radiotherapy.

**Results:**

Twenty evaluable patients comprised the Arm A feasibility cohort. No protocol-defined treatment-limiting toxicities occurred (0 of 20 patients; exact 95% CI, 0.0%-16.8%). Grade 3 or higher adverse events occurred in 12 patients (60%), predominantly hematologic toxicity attributable to chemoradiotherapy; lymphopenia occurred in 6 patients (30%). Median usage through visit 4 was 76.5%, and median time to first attainment of at least 75% daily use was 2 days. Median progression-free survival was 6.9 months (95% CI, 2.7–14.0), and median overall survival was 18.0 months (95% CI, 12.1–19.9).

**Conclusions:**

Initiation of TTFields before and during radiotherapy was feasible and was not associated with protocol-defined treatment-limiting toxicity in the Arm A cohort. Because the TLT definition was narrow and efficacy analyses were exploratory and exposure-conditioned, these findings should be interpreted as feasibility data rather than evidence of improved tumor control or survival. The results support randomized evaluation of earlier TTFields integration in newly diagnosed glioblastoma.

**Trial registration:**

German Clinical Trials Register DRKS00016667, registered 26 February 2019.

**Supplementary Information:**

The online version contains supplementary material available at 10.1186/s12885-026-16613-y.

## Background

Glioblastoma, isocitrate dehydrogenase (IDH) wildtype, is the most common malignant primary brain tumor in adults and is classified as a WHO grade 4 adult-type diffuse glioma in the 2021 WHO Classification [1]. Its age-adjusted incidence is ~ 3–5 per 100,000 in Europe and 2.5 per 100,000 for grade 4 IDH-wildtype astrocytoma/glioblastoma in the United States [2, 3]. Standard management remains maximal safe resection followed by 60 Gy radiotherapy with concomitant and adjuvant temozolomide, yet median overall survival in pivotal trials remains ~ 14–16 months and is influenced by MGMT promoter methylation and extent of resection [4–6]. For MGMT-methylated tumors, lomustine-temozolomide (CeTeG/NOA-09) is used in selected European centers [7].

Tumor Treating Fields (TTFields) are an established additional modality during maintenance temozolomide based on EF-14, in which TTFields initiated after completion of chemoradiotherapy improved progression-free survival and overall survival versus temozolomide alone with mainly grade 1–2 dermatologic toxicity [8, 9]. TTFields exert anti-mitotic effects through intermediate-frequency alternating fields delivered via scalp arrays [10, 11]. Preclinical data further support radiosensitization via interference with DNA damage responses, including delayed double-strand break repair and impaired homologous recombination [11, 12]. Early clinical and dosimetric studies indicate feasibility of concurrent TTFields with radiotherapy, with preserved deep target coverage and a potential increase in scalp dose if arrays remain in place, supporting array removal during irradiation [13–15]. Another biological rationale for earlier initiation is the significant rate of measurable tumor regrowth between early postoperative and pre-radiotherapy MRI ("rapid early progression"), which is frequent and prognostically adverse [16–19]. PriCoTTF was therefore designed to test whether initiating TTFields before and during chemoradiotherapy is feasible and can be delivered without protocol-defined treatment-limiting toxicity in newly diagnosed glioblastoma, extending prior single-center feasibility data and informing ongoing randomized evaluation in EF-32/TRIDENT (NCT04471844) [15, 20]. This report presents results from Arm A only (patients < = 70 years receiving standard 6-week chemoradiotherapy with concurrent TTFields). The results from Arm B (elderly patients) will be reported separately.

## Methods

### Study design and oversight

**Fig. 1:**
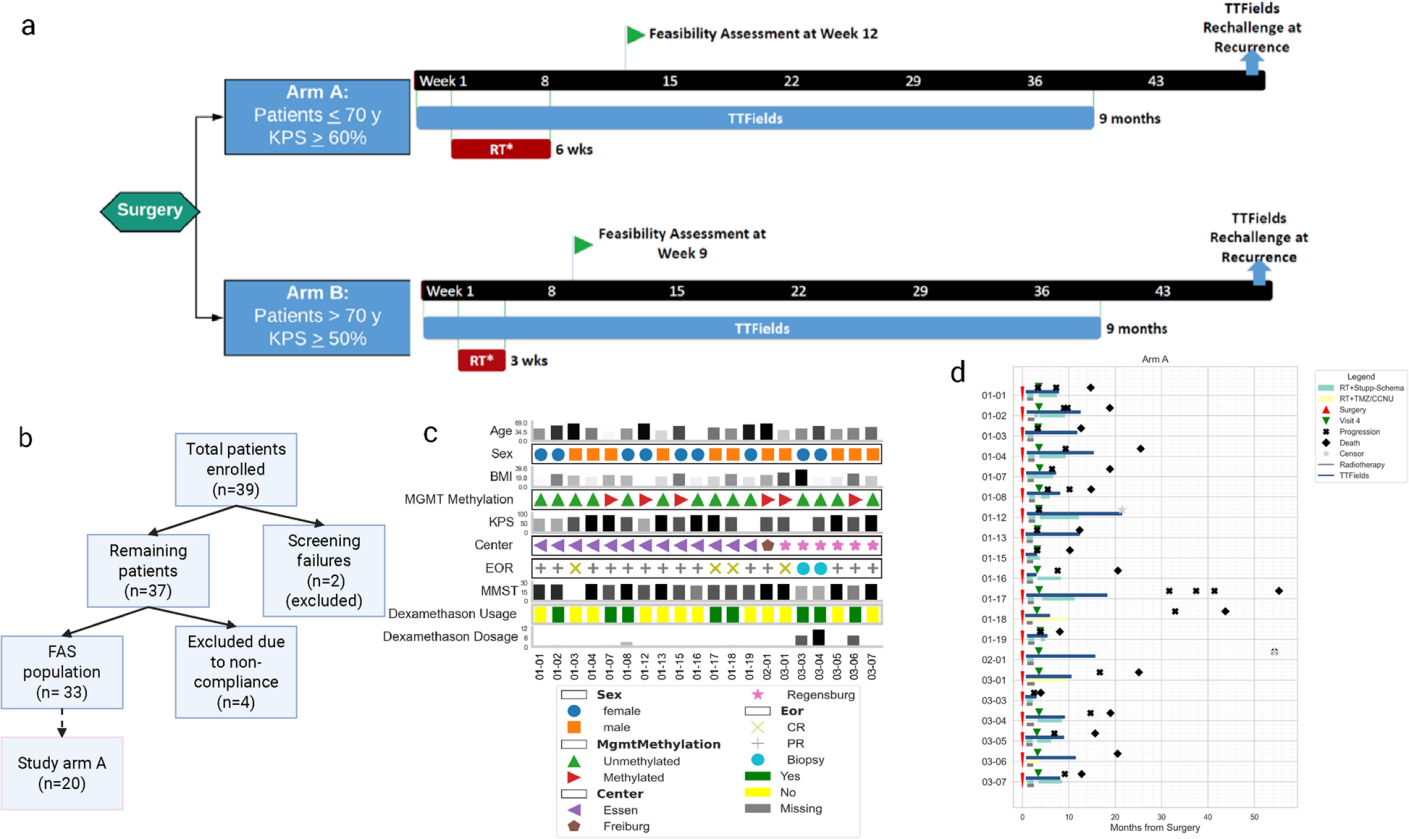
Trial Design, Participant Flow, Baseline Overview, and Patient-Level Treatment Timelines. Panel A shows the protocol schema. Arm A enrolled patients 70 years of age or younger with a Karnofsky Performance Status of at least 60 and planned standard chemoradiotherapy. Feasibility was assessed at week 12. Arm B enrolled patients older than 70 years with a Karnofsky Performance Status of at least 50 and planned hypofractionated radiotherapy. Feasibility was assessed at week 9. In both arms, Tumor Treating Fields were initiated 2 to 4 weeks after surgery and 1 to 2 weeks before radiotherapy and were planned to continue for 9 months. Tumor Treating Fields rechallenge at recurrence was permitted by protocol. Panel B shows the study flow for the reported cohort. 39 patients were screened, two screening failures occurred before treatment initiation, 37 patients entered the overall Safety Analysis Set, and 33 patients comprised the overall Full Analysis Set after exclusion for insufficient compliance. In Arm A specifically, 23 patients entered the Arm A Safety Analysis Set and 20 comprised the exposure-conditioned Arm A Full Analysis Set after three Arm A patients were excluded for insufficient TTFields exposure. This 20-patient Arm A cohort is represented in the present feasibility and exploratory survival analyses. Panel C summarizes baseline characteristics of the 20 patients in Arm A as a patient-level heat map. Continuous variables are displayed on graded scales (age, body-mass index, Karnofsky Performance Status, MMSE score, and dexamethasone dose), and categorical variables are shown by symbols and colors for sex, O6-methylguanine-DNA methyltransferase promoter methylation status, center, extent of resection, and dexamethasone use. Panel D shows patient-level treatment timelines from surgery. Horizontal bars indicate exposure to Tumor Treating Fields, radiotherapy, and post-radiotherapy systemic therapies. Colored segments denote individual drug classes or regimens as indicated in the key. Symbols denote surgery, study visits, progression, death, and censoring. The abbreviations are CR for complete resection and PR for partial resection

We conducted a prospective, open-label, nonrandomized, multicenter phase I/II trial. The trial tested whether TTFields can be started before and given during chemoradiotherapy in patients with newly diagnosed IDH-wildtype glioblastoma. Arm A enrolled patients 70 years of age or younger with a Karnofsky Performance Status (KPS) of at least 60%. Patients received standard radiotherapy for 6 weeks. Arm B enrolled patients older than 70 years with a KPS of at least 50%. Patients received 3-week hypofractionated radiotherapy. In both arms, TTFields started early after surgery and continued during chemoradiotherapy and the next 9 months. Feasibility assessments were prespecified at week 12 (Arm A) and week 9 (Arm B). The protocol allowed TTFields rechallenge at recurrence (Fig. 1a). The trial was registered before enrollment (German Clinical Trials Register, DRKS00016667; registered February 26, 2019). The device investigation is listed in EUDAMED (CIV-18–08–025247). The first patient was enrolled on June 25, 2019. The study followed the Declaration of Helsinki and ICH-GCP E6(R2). The coordinating center ethics committee (Universität Duisburg-Essen) approved the trial (vote 18–8316-MF; February 6, 2019). All sites obtained local approvals (listed in the Supplementary Clinical Trial Protocol). All patients gave written informed consent, including consent for device log-file collection and de-identified data sharing. University Hospital Essen was the legal sponsor. NovoTTF-200A (Optune Gio) was supplied by Novocure GmbH. Novocure had no role in the trial design, data analysis, data interpretation, or manuscript writing. Device Support Specialists provided routine device maintenance and extracted log files as specified in the protocol. They did not conduct the study and did not assess endpoints. All authors had full access to the data. The corresponding author made the final decision to submit the manuscript. The study was completed on August 15, 2023. This manuscript adheres to the applicable CONSORT 2010 reporting items. Items concerning randomization, allocation concealment, and blinding are not applicable because Arm A was a nonrandomized feasibility cohort. A completed CONSORT 2010 checklist is provided as an additional file.

### Participants (Arm A)

Eligible patients were 18 to 70 years of age. They had newly diagnosed glioblastoma or gliosarcoma confirmed by histology and molecular testing per WHO CNS5 [1]. Local neuropathology institutions verified diagnoses; central pathology review was not performed. IDH-wildtype status was tested by immunohistochemistry (anti-IDH1 R132H, clone H09, Dianova). For non-R132H cases, sequencing was performed per standard WHO CNS5 workflows. Patients needed a KPS of at least 60% and an expected survival of at least 3 months. Patients had undergone resection or biopsy. Extent of resection was not an eligibility criterion, but it was recorded and analyzed as a prognostic variable. Patients were planned to receive focal radiotherapy to 60 Gy in 30 fractions. All patients treated per the Stupp regimen received concomitant temozolomide during radiotherapy. Patients with MGMT-methylated tumors were eligible to receive lomustine-temozolomide (CeTeG/NOA-09). Patients treated with this regimen did not receive concomitant temozolomide during radiotherapy, consistent with the CeTeG regimen. Key exclusion criteria followed the Optune Gio Instructions for Use (edition in force at trial start: 2018). Exclusions included active implanted devices (e.g., pacemakers, defibrillators, programmable shunts), untreated or unstable skull defects, and hypersensitivity to system components (e.g., conductive hydrogels). Clinically significant arrhythmias were excluded by protocol medical criteria (e.g., atrial fibrillation with hemodynamic compromise; ventricular arrhythmias requiring antiarrhythmic therapy). Additional exclusions included pregnancy or breastfeeding, a positive pregnancy test at screening, failure to follow contraception requirements, or use of investigational agents within 30 days before enrollment.

### Interventions

TTFields started 2 to 4 weeks after surgery and 1 to 2 weeks before chemoradiotherapy and were delivered at 200 kHz using Optune Gio. Clinical dosing was defined by daily wear time (usage). Field intensity depended on array layout and patient anatomy. Arrays were placed on a shaved scalp using individualized NovoTal mapping. Arrays were changed every 3 to 4 days. All handling followed the manufacturer's Instructions for Use. Skin toxicity prevention and management followed the recommendations by Lacouture et al. [21]. Measures included education of patients and caregivers, strict hand hygiene before array changes, rotation of array positions by about 2 cm, avoiding ceramic discs over scars, screws, or wires, and ensuring ventilation when arrays were covered. TTFields were held for CTCAE v5.0 grade 3 or higher moist desquamation and restarted only after improvement to grade 1. Radiotherapy continued unless moist desquamation exceeded 25 cm2. During each radiotherapy fraction, the device was switched off, the arrays, however, were not removed; regular array-position shifts were used to mitigate skin exposure and were consistent with simulation data suggesting that minor dosimetric effects from arrays in place can be offset by array repositioning. This aimed to minimize skin irritation from daily removal and followed prior feasibility and dosimetry work [13–15]. Radiotherapy used MRI/CT fusion and image-guided IMRT or VMAT. The dose was 60 Gy in 30 fractions (2 Gy per fraction). The clinical target volume included the resection cavity and any residual enhancing tumor with a 2.0 cm anatomically constrained margin. The planning target volume expanded the clinical target volume by 2 to 5 mm. Dose constraints for optic nerves, optic chiasm, and brainstem followed protocol-defined institutional organ-at-risk guidelines. TTFields continued for at least 9 months and overlapped with adjuvant chemotherapy. TTFields were stopped at radiographic or clinical progression, unacceptable toxicity, or patient withdrawal. TTFields rechallenge was allowed after at least 4 weeks off TTFields or after full recovery from TTFields-related skin toxicity. Chemotherapy was assigned by MGMT promoter methylation status. Assessment of methylation status was performed according to validated institutional standards. Patients with MGMT-unmethylated tumors received concomitant temozolomide (75 mg/m2) during radiotherapy, then adjuvant temozolomide (150–200 mg/m2 on days 1–5 every 28 days for six cycles) per the Stupp regimen [4]. Patients with MGMT-methylated tumors were eligible to receive lomustine (100 mg/m2 on day 1) plus temozolomide (100–200 mg/m2 on days 2–6) every 6 weeks per CeTeG/NOA-09. Patients treated with this regimen did not receive concomitant daily temozolomide during radiotherapy. Dose changes and hematologic toxicity management followed institutional standards. Patients were instructed to use TTFields at least 75% of each day (about > = 18 h/day). This target was prespecified and based on prior evidence linking higher usage with better survival [22]. TTFields usage was recorded by device logs with 1-min resolution. Logs were encrypted, tamper-protected, and sent via a secure channel to the manufacturer's server in a regulatory-compliant environment. A Novocure Device Support Specialist extracted technical data monthly and checked log completeness per protocol. Novocure remained blinded to clinical outcomes and did not collect clinical data, analyze clinical outcomes, or assess endpoints. Sites kept full oversight of clinical data. An audit trail with timestamps and access logs was maintained. Sites checked data accuracy before database lock.

### Outcomes

The primary endpoint was the incidence of treatment-limiting toxicities (TLTs) from TTFields start until 4 weeks after radiotherapy (visit 4), as prespecified (Supplementary material. PriCoTTF Protocol Version 5.0). A TLT was any of the following events. Grade 4 radiation dermatitis was a TLT. Grade 3 dermatitis with moist desquamation > = 25 cm2 leading to a compliance rate below 50% was a TLT. Grade 3 dermatitis with moist desquamation > 1 cm beneath a transducer contact point leading to a compliance rate below 50% despite skin protection measures was a TLT. Any other toxicity expected to be related to the combined therapy leading to a compliance rate below 50% until the primary endpoint was also a TLT. This endpoint was designed to test feasibility and treatment-limiting toxicity affecting device continuation. It was not intended to capture all clinically meaningful toxicity from chemoradiotherapy, TTFields, or their combination. Causality was assessed in two steps. First, the treating investigator assessed causality. Second, the Sponsor Delegated Person performed an assessment, while an independent Data Monitoring Safety Committee (medical statistician, radiation oncologist, neuro-oncologist) provided safety oversight. The committee was independent of the sponsor. Secondary endpoints were overall survival (OS) and progression-free survival (PFS). OS was from surgery to death from any cause. PFS was from surgery to radiologic progression or death. Treatment-related assessments used the postoperative, pre-radiotherapy baseline visit. Survival analyses used surgery as time zero. Progression was assessed by site investigators using RANO criteria [23]. MRI included contrast-enhanced 3D T1 MPRAGE, T2, FLAIR, and diffusion-weighted imaging at baseline, after radiotherapy, and every 8 to 12 weeks. The same scanner and protocol were used when feasible. Corticosteroid dose was recorded at each scan. TTFields use was calculated from encrypted logs as the proportion of active device time per 24 h, averaged over all treatment days. Adverse events were coded with MedDRA v25.1 and graded by CTCAE v5.0. Serious adverse events were managed and reported per MDR, DIN EN ISO 14155, and applicable pharmacovigilance standards.

### Visit schedule

Visits followed the PriCoTTF schedule: baseline (2–4 weeks after surgery), immediately before radiotherapy (V1), weekly during radiotherapy (V2), at radiotherapy completion (V3), and 4 weeks after radiotherapy (V4). Core assessments included neurologic examination, KPS, adverse events, laboratory tests, and device checks. Quality of life (EORTC QLQ-C30/BN20) and cognition (MMSE) were collected at baseline, V3, and V4. They were not collected during weekly V2 visits to reduce burden. V2 visits focused on safety and TTFields-related skin monitoring. The full schedule of activities is in the protocol (Version 5.0, Sect. 2) and summarized in Supplementary Fig. 1.

### Statistical analysis

In Arm A, the primary endpoint was evaluated with Simon's optimal two-stage design. Stage 1 enrolled 7 patients. If 3 or more had a TLT by 4 weeks after radiotherapy, the regimen was considered not feasible and accrual stopped. Otherwise, 13 more patients were enrolled (total 20). The design tested a null TLT rate of at least 40% versus an alternative of 15% or less, with one-sided alpha 0.05 and 80% power. The regimen was considered feasible if 4 or fewer of 20 patients had a TLT. Exact binomial 95% confidence intervals (Clopper-Pearson) were calculated. The two-stage rule is shown in Supplementary Fig. 2. The Arm A Safety Analysis Set (SAF) included 23 patients who started TTFields and radiotherapy. OS and PFS analyses were performed in an exposure-conditioned Arm A Full Analysis Set (FAS) of 20 patients, after excluding three Arm A SAF patients for insufficient TTFields exposure (01–14, 06–01, 06–03), operationalized in this analysis as average documented usage below the 50% compliance threshold before the primary endpoint. This threshold was used because OS and PFS analyses were intended to contextualize outcomes among patients who had received sufficient early device exposure. These analyses were not intention-to-treat efficacy analyses. Across the full trial, the FAS comprised 33 patients. The present manuscript reports only the Arm A cohort. Continuous variables are reported as median (IQR). Categorical variables are counts and percentages, with denominators specified in the text, tables, and figure legends. Survival curves were estimated with Kaplan–Meier methods. Group comparisons used two-sided log-rank tests. Median follow-up was calculated with the reverse Kaplan–Meier method. Cox proportional-hazards models assessed associations of age, MGMT status, extent of resection, and KPS with OS and PFS. Proportionality was checked with Schoenfeld residuals. No formal survival power calculation was performed because the sample size was determined by the Simon feasibility design rather than an efficacy hypothesis. All survival analyses are exploratory and descriptive. Because analyses were exploratory and FAS-conditioned, two-sided P values were not adjusted for multiplicity and are descriptive. Longitudinal TTFields usage (percent "on-time" per 24 h) was modeled from treatment start using a linear mixed-effects model with random patient intercepts and visit as a fixed effect (MixedLM, statsmodels). Predicted mean usage over the first 12 weeks defined usage tertiles for exploratory plots. The prespecified usage target (> = 75%, approximately 18 h/day) was also tested as a clinically interpretable cutoff in sensitivity analyses. Analyses used Python 3.12.9 with lifelines v0.28.0, statsmodels v0.14.1, pingouin v0.5.4, scipy v1.12.0, numpy v1.26.4, pandas v2.2.1, and matplotlib v3.8.2. Key survival estimates were cross-validated in R using the survival package.

## Results

### Patient enrollment and treatment overview

A total of 39 patients were screened, of whom two did not meet eligibility criteria before treatment initiation (Fig. 1b). The overall Safety Analysis Set (SAF) comprised 37 patients who started both TTFields and radiotherapy, consistent with the protocol-defined safety population. For exploratory survival analyses, the overall Full Analysis Set (FAS) comprised 33 patients who initiated TTFields and demonstrated sufficient compliance. In the Arm A cohort reported here, the SAF comprised 23 patients. Three Arm A SAF patients had insufficient TTFields exposure according to the analysis threshold and remained in the SAF but were excluded from the exposure-conditioned FAS, yielding 20 Arm A FAS patients for exploratory OS and PFS analyses.

As a result, all OS and PFS outcomes reported for the primary Arm A analysis reflect an exposure-conditioned, non-intention-to-treat population and may underestimate event rates relative to a strict intention-to-treat analysis. Because this report focuses on the concurrent TTFields-radiotherapy feasibility cohort, efficacy results correspond to the 20 Arm A patients aged < = 70 years with KPS > = 60% in the exposure-conditioned FAS, using surgery as the prespecified time origin for survival modeling. The broader Arm A SAF (n = 23) is retained for sensitivity analyses of OS and PFS. This population structure (overall SAF, Arm A SAF, and Arm A exposure-conditioned FAS) is used throughout the manuscript.

### Baseline characteristics

Baseline demographics and clinical characteristics for the 20 patients in Arm A are shown in Fig. 1c and Table 1. Median age was 53.5 years (range 38–69), and 11 patients (55.0%) were male. The median body-mass index was 25.6 kg/m2 (range 21.2–39.6). MGMT promoter methylation was present in 6 of 20 patients (30.0%), with 14 (70.0%) unmethylated. Functional status was relatively preserved, with a median KPS of 90 (range 70–100). Extent of resection consisted of complete resection (CR) in four patients (20.0%), partial resection (PR) in 14 (70.0%), and biopsy in 2 (10.0%). Cognitive performance by Mini-Mental State Examination (MMSE) showed a median score of 28 (range 24–30). At baseline, eight patients (40.0%) were receiving dexamethasone, with a median daily dose of 3 mg (range 1–12 mg). First-line treatment courses for all 20 evaluable Arm A patients are summarized in Fig. 1d. RT + Stupp-Schema was the predominant first-line regimen, administered in 17 of 20 patients (85.0%), while RT + TMZ/lomustine was given in 3 of 20 patients (15.0%).

**Table 1:** Baseline characteristics of Arm A and EF-14 TTFields cohorts

**Characteristic**	**Arm A (***n*** = 20)**	**EF-14 TTFields arm (***n*** = 466)**
Age, years	Median 53.5 (range 38–69)	Median 56 (range 19–83)
Sex	Male 11 (55.0%); female 9 (45.0%)	Male 316 (68%); female 150 (32%)
BMI	Median 25.6 (range 21.2–39.6)	Not reported
MGMT promoter methylation	Unmethylated 14 (70.0%); methylated 6 (30.0%)	Unmethylated 137 (29%); methylated 209 (45%); not available/invalid 120 (26%)
KPS	Median 90 (range 70–100)	Median 90 (range 60–100); missing 4
Center	Essen 13 (65.0%); Regensburg 6 (30.0%); Freiburg 1 (5.0%)	USA 221 (47%); outside USA 245 (53%)
Extent of resection	Partial resection 14 (70.0%); complete resection 4 (20.0%); biopsy 2 (10.0%)	Partial resection 157 (34%); complete resection 249 (53%); biopsy 60 (13%)
MMSE	Median 28 (range 24–30)	Median 28 (range 24–30)
Dexamethasone use	No 12 (60.0%); yes 8 (40.0%)	Not reported
Dexamethasone dose	Median 3.0 mg (range 1.0–12.0) among patients receiving dexamethasone	Not reported
First-line therapy	RT plus Stupp regimen 17 (85.0%); RT plus temozolomide/lomustine 3 (15.0%)	RT plus Stupp regimen 466 (100%)

Values are medians with ranges or numbers of patients with percentages. EF-14 data are shown for descriptive context only; MGMT status, extent of resection, eligibility, and time zero for progression-free survival differ between cohorts. *Abbreviations*: *BMI* body-mass index, *CR* complete resection, *KPS* Karnofsky Performance Status, *MGMT* O6-methylguanine-DNA methyltransferase, *MMSE* Mini-Mental State Examination, *PR* partial resection, *RT* radiotherapy, *TTFields* Tumor Treating Fields

### Safety and adverse events

**Fig. 2:**
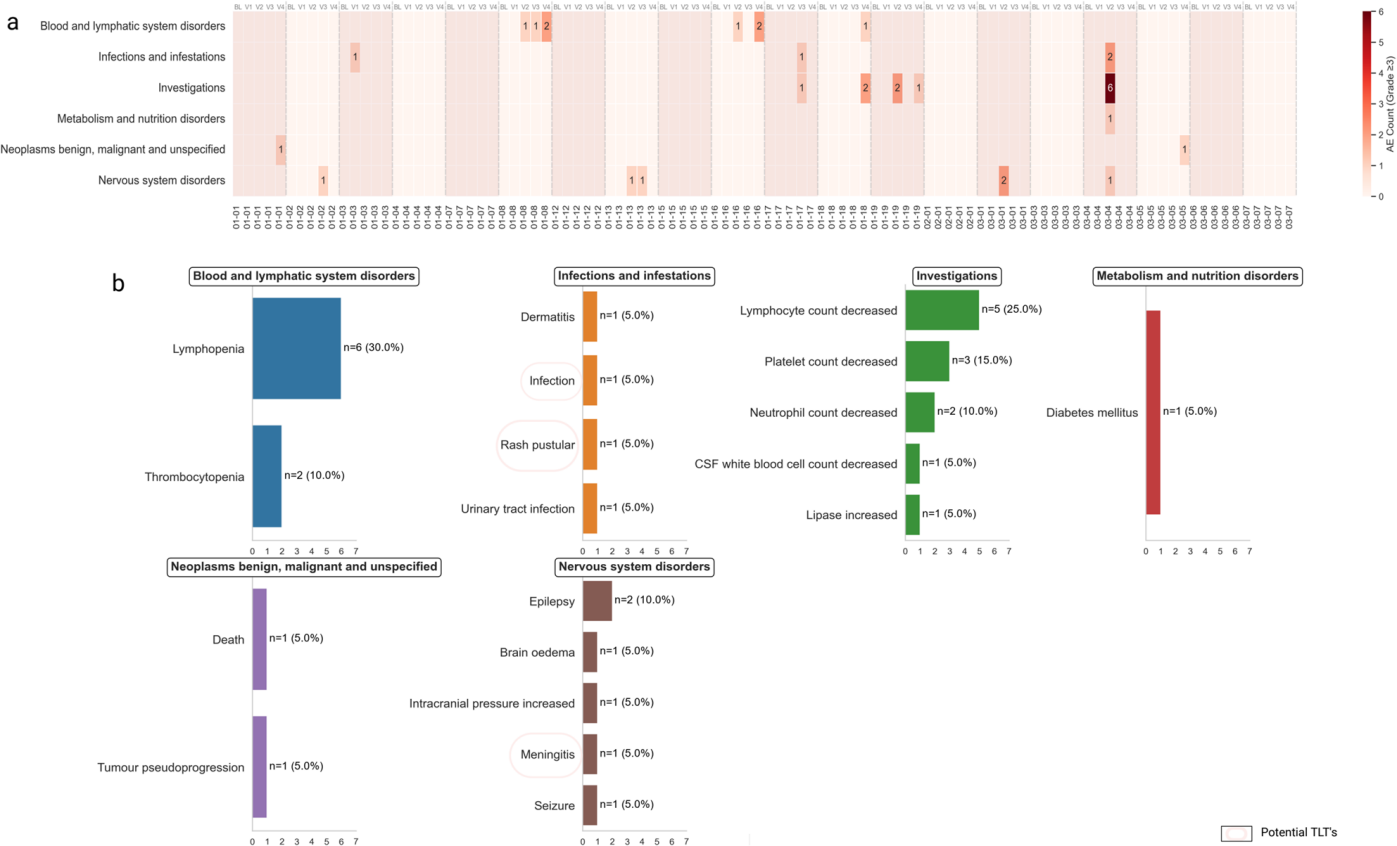
Safety and Grade 3 or Higher Adverse Events in Arm A. Panel A shows a patient-by-visit heat map of grade 3 or higher adverse events from baseline through Visit 4, grouped by Medical Dictionary for Regulatory Activities system organ class. Columns are organized by patient and study visit; color intensity represents the number of adverse events recorded in a given cell. Open outlines identify events reviewed as potential treatment-limiting toxicities. Panel B summarizes grade 3 or higher adverse events by preferred term within each system organ class. Bars indicate the number of patients with at least one event; percentages are patient-level proportions among the 20 patients in Arm A. Event counts therefore reflect affected patients rather than total event episodes. The three serious adverse events reviewed by the independent safety committee were pustular rash, meningitis, and systemic infection; none fulfilled the prespecified definition of treatment-limiting toxicity

Safety outcomes for the 20 evaluable Arm A patients are summarized in Fig. 2. A total of 33 Grade > = 3 adverse events (AEs) were reported across 12 of 20 patients (60%). Event counts refer to individual AEs, whereas percentages refer to the number of patients affected. No protocol-defined TLTs occurred (0/20 patients), consistent with the feasibility criterion of < = 4 TLTs in the Simon two-stage design. The most frequently involved system organ classes were Blood and Lymphatic Disorders, Investigations, Nervous System Disorders, and Infections and Infestations. Common Toxicity Criteria (CTC) Grade > = 3 lymphopenia occurred in six of all 20 evaluable Arm A patients (30%), and typically arose during or shortly after radiotherapy. This rate is consistent with the 25–40% incidence of grade 3–4 lymphopenia reported under standard chemoradiotherapy per the Stupp protocol [4, 24], suggesting no clinically meaningful additive hematologic effect of concurrent TTFields. All hematologic events were managed with supportive care and close monitoring, without requiring modification of radiotherapy (RT) or TTFields. Dermatologic toxicity was monitored at each device-focused visit because the TLT definition was closely linked to skin toxicity and device continuation. Any-grade dermatologic adverse events occurred in 18 of 20 patients (90%) and were predominantly grade 1 or 2. Three grade 3 dermatologic events occurred in two patients (10%). No grade 4 dermatologic event, no grade 3 moist desquamation fulfilling the protocol TLT criteria, and no permanent TTFields discontinuation for skin toxicity occurred by the primary endpoint. A detailed event-level breakdown is provided in Supplementary Table 5.

Three serious adverse events (SAEs) were reviewed by the independent safety committee. The events were a pustular rash (Patient 01–17, V3, Grade 3) at the end of radiotherapy, meningitis (Patient 03–01, V2, Grade 3) during radiotherapy, and systemic infection (Patient 03–04, V2, Grade 3) during the concurrent TTFields + RT period. Each resolved with standard therapy, topical or systemic antibiotics, or supportive care. No patient required permanent TTFields discontinuation. The committee determined that none met the prespecified causality criteria for TTFields + RT-related toxicity. The rash occurred outside the array contact field and lacked thermal features. Meningitis and systemic infection were judged unrelated to localized scalp or radiation effects. Patient-reported dermatologic burden was captured separately by the EORTC BN20 itchy-skin domain, which worsened significantly during early treatment as reported below and in Fig. 4e and Supplementary Fig. 7.

Accordingly, the feasibility endpoint was achieved, with an observed TLT rate of 0% (exact 95% CI 0.0–16.8%). A conservative sensitivity scenario counting the three SAEs as potential TLTs yielded an estimated rate of 0.150 (3/20, 95% CI, 0.032–0.379), which remained below the prespecified 40% threshold (one-sided exact binomial *P* = 0.016). A further sensitivity analysis counting any grade 3 or higher dermatologic event as a TLT yielded an estimated rate of 0.100 (2/20, 95% CI, 0.012–0.317). These results are shown in Supplementary Table 1.

### Tumor treating fields usage patterns

**Fig. 3:**
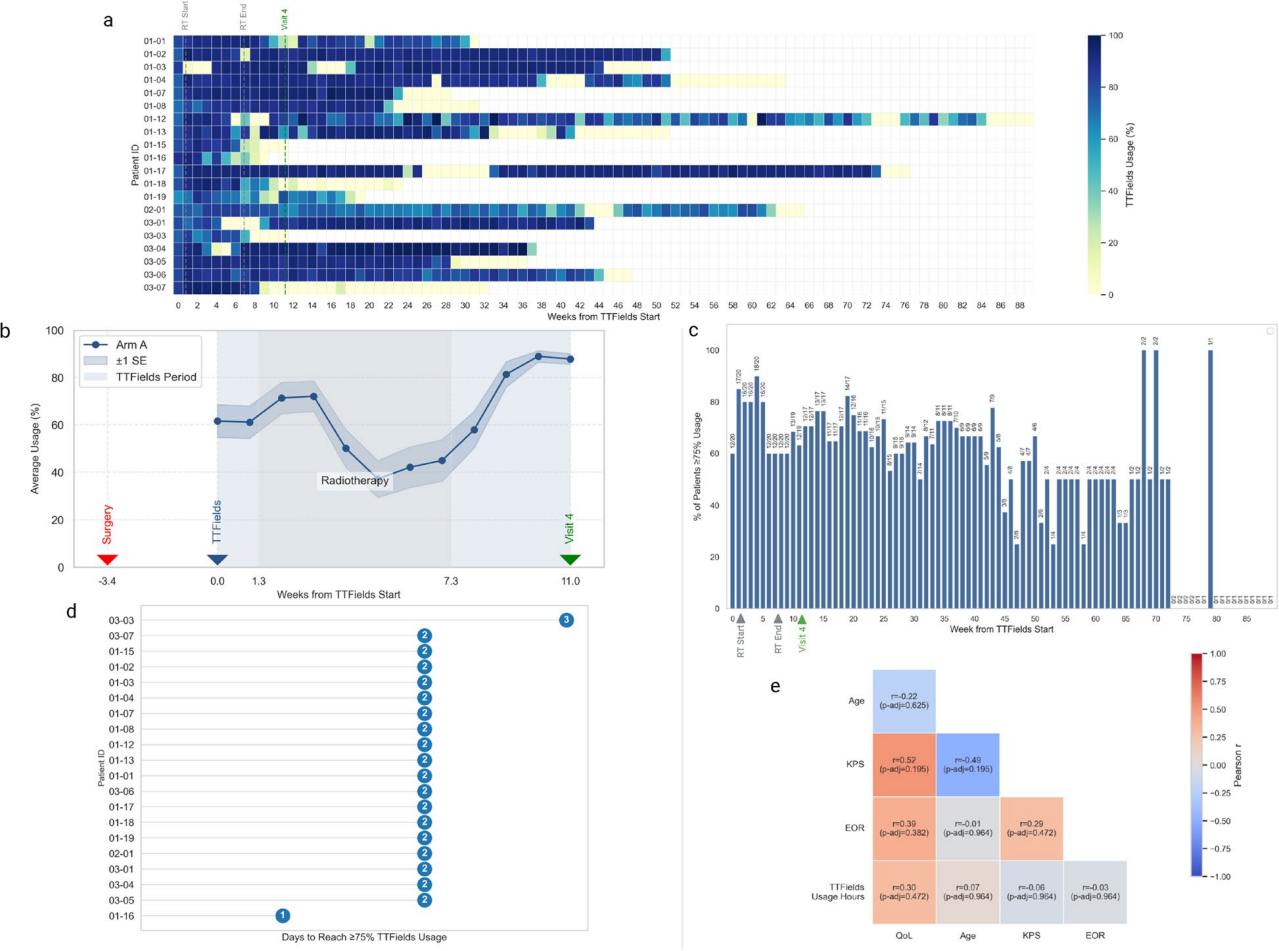
Tumor Treating Fields Usage Patterns and Exploratory Correlates in Arm A. Panel A shows a longitudinal heat map of daily Tumor Treating Fields usage, expressed as the percentage of each 24-h period during which the device was active for each patient from treatment initiation onward. Panel B shows weekly mean usage with standard-error bands; vertical markers indicate surgery, Tumor Treating Fields initiation, the radiotherapy interval, and visit 4. Panel C shows the weekly proportion of patients meeting the prespecified usage threshold of at least 75% daily use. Values above the bars indicate the percentage meeting that threshold in each week. Panel D shows the number of days required for each patient to first achieve at least 75% daily use; the cohort median was 2 days. Panel E shows Pearson correlation coefficients for the relationships among age, Karnofsky Performance Status, extent of resection, cumulative Tumor Treating Fields usage, and quality of life measures; values in the matrix are Pearson r coefficients with false-discovery-rate-adjusted P values. Abbreviation: QoL denotes quality of life

TTFields usage in Arm A was high. The overall median usage until visit 4 was 76.5%, with per patient averages provided in Supplementary Table 2, and usage showed a transient decline during radiotherapy. The apparent post-radiotherapy increase should be interpreted descriptively because patients with poor usage during radiotherapy were less represented after radiotherapy. Daily device-on heatmaps (Fig. 3a) and individual trajectories (Supplementary Fig. 3) demonstrated consistently high usage for most patients, with only short interruptions around radiotherapy start and end. Cohort-level mean usage declined during radiotherapy (mean 53.7%), indicating that the concurrent phase poses the greatest barrier to sustained device use. This during-radiotherapy mean was below the prespecified 75% daily usage target derived from prior maintenance-phase TTFields experience, and it therefore provides feasibility information rather than evidence that radiosensitizing exposure was consistently achieved during the entire radiation window. Mean usage increased after radiotherapy (mean 78.6%), reaching 89.6% by visit 4 (Fig. 3b). Median values remained substantially higher than means throughout, reflecting a right-skewed distribution dominated by high-usage individuals, whereas lower-usage days from a minority of patients pulled down the cohort means (Supplementary Fig. 4).

The median time to first attainment of > = 75% daily TTFields usage was 2 days (range 1–3) (Fig. 3d). Across weeks 0–12, the median weekly proportion of patients achieving > = 75% usage was 68.4%, and it was 68.8% across weeks 0–24 (Fig. 3c). Exploratory correlations (Fig. 3e) showed no significant association between TTFields usage and age, KPS, extent of resection (all false discovery rate (FDR)-adjusted p > = 0.05). Overall, these data demonstrate rapid onboarding and sustained usage across concurrent and adjuvant treatment in Arm A.

### Functional status, cognitive outcomes, and quality of life

**Fig. 4:**
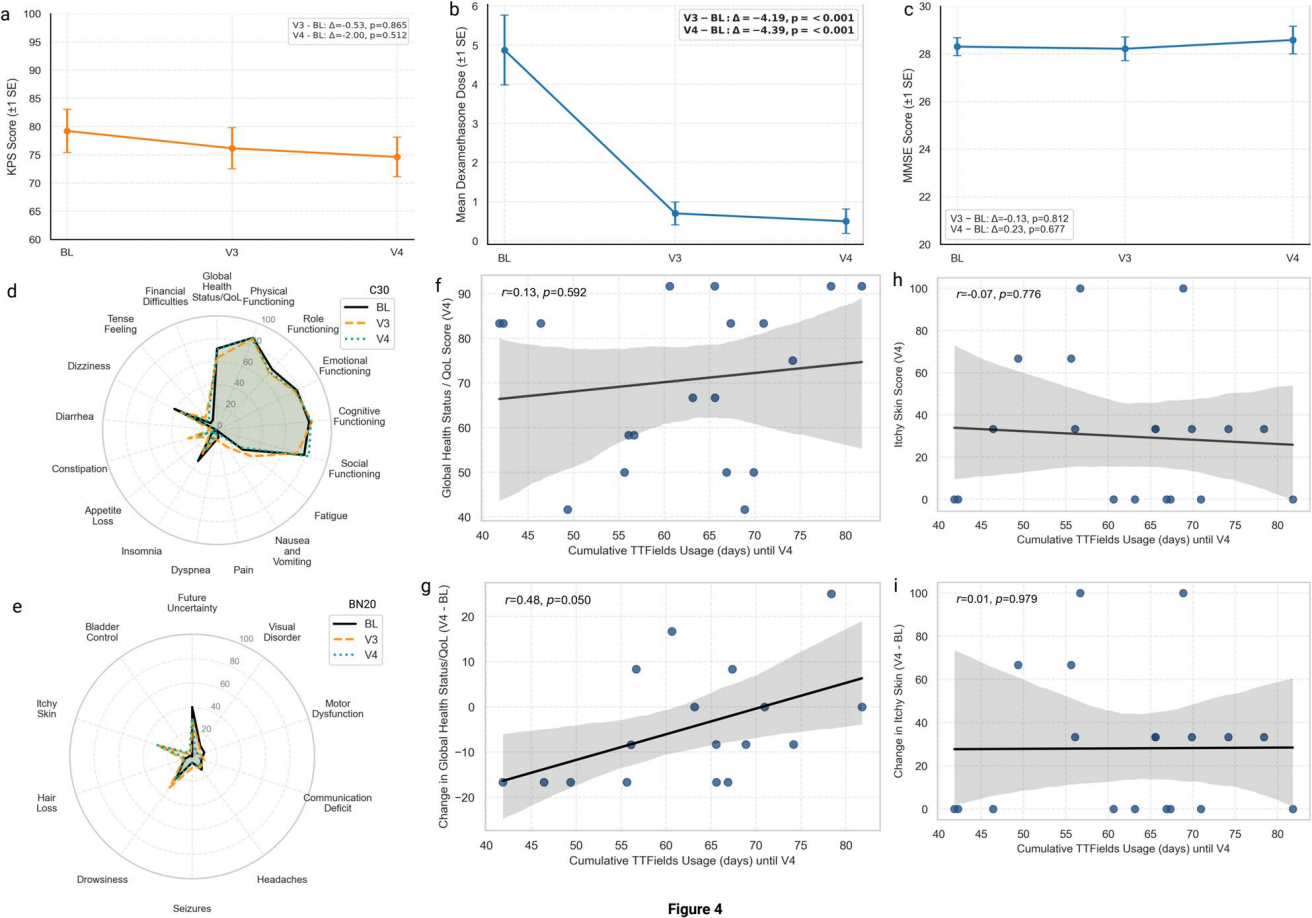
Functional, Cognitive, and Quality-of-Life Outcomes During Early Tumor Treating Fields Therapy. Panels A through C show longitudinal assessments at baseline, at the end of radiotherapy (visit 3), and 4 weeks after radiotherapy (visit 4). Panel A shows the mean Karnofsky Performance Status score with standard errors. Panel B shows the mean daily dexamethasone dose with standard errors. Panel C shows Mini-Mental State Examination scores with reference lines at scores of 24 and 18, indicating thresholds commonly used for mild and moderate cognitive impairment. Panels D and E show radar plots of group-level patient-reported outcomes from the European Organisation for Research and Treatment of Cancer Quality of Life Questionnaire-Core 30 (Panel D) and brain tumor module BN20 (Panel E). For functional scales, higher scores indicate better functioning; for symptom scales, higher scores indicate greater symptom burden. Panels F through I show exploratory scatterplots relating cumulative Tumor Treating Fields exposure through visit 4 to global health status at visit 4, change in global health status from baseline to visit 4, itchy skin score at visit 4, and change in itchy skin score from baseline to visit 4. Solid lines indicate simple linear fits and shaded bands indicate 95% confidence intervals

Functional status, cognitive performance, and corticosteroid use during TTFields therapy are summarized in Fig. 4 and Supplementary Figs. 5–7. A linear mixed-effects model was employed to account for repeated measures over time. KPS scores remained stable. Changes from baseline were not significant at visit 3 (Δ = −0.53, *p* = 0.865) or visit 4 (Δ = −2.00, *p* = 0.512). These results are shown in Fig. 4a and Supplementary Fig. 5a. Mean dexamethasone dose declined substantially. Significant reductions were observed from baseline to visit 3 (Δ = −4.19 mg, *p* < 0.001) and visit 4 (Δ = −4.39 mg, *p* < 0.001). These results are shown in Fig. 4b and Supplementary Fig. 5b. This reduction likely reflects the natural resolution of postoperative edema in addition to any treatment-related effect and cannot be causally attributed to TTFields in the absence of a control group. To avoid overstating this finding, corticosteroid reduction is treated as a descriptive supportive observation and is not included as an efficacy claim in the Abstract or Conclusions. MMSE scores remained preserved throughout therapy. Differences from baseline were not significant at visit 3 (Δ = −0.13, *p* = 0.812) or visit 4 (Δ = 0.23, *p* = 0.677). These results are shown in Fig. 4c and Supplementary Fig. 5c.

Global health status and functional outcomes assessed via the EORTC QLQ-C30 remained largely stable throughout the treatment period (Fig. 4d), with no significant deterioration observed in core domains including physical, role, emotional, and cognitive functioning (Supplementary Fig. 6). Although global health status at visit 3 showed a nominal decline from baseline (Δ = −9.32, *p* = 0.037), the effect was not statistically significant after correction for multiple testing (pₐ*dj* = 0.190). Global health status returned to baseline levels at visit 4. Several symptom domains such as fatigue, insomnia, and financial difficulties exhibited mild worsening, but none reached statistical significance. In contrast, a significant increase in constipation was observed at visit 3 compared to baseline (Δ = 22.74, pₐ*dj* = 0.025), which resolved by visit 4 (pₐ*dj* = 0.610). Similarly, nausea and vomiting showed a statistically significant increase at visit 3 (pₐ*dj* = 0.018). Analysis of the brain tumor-specific QLQ-BN20 module (Fig. 4e, Supplementary Fig. 7) revealed low overall symptom burden, with itchy skin being the only domain showing consistent worsening over time (BL-V3: Δ = 27.95, pₐ*dj* = 0.002; BL-V4: Δ = 28.16, pₐ*dj* = 0.002). Overall, quality of life measures were preserved, and dermatologic symptoms (itchy skin) showed the main measurable worsening.

### Correlation between tumor treating fields usage and itching of the skin

Correlation analysis showed no significant association between cumulative TTFields usage and global health status at visit 4 (r = 0.13, *p* = 0.592; Fig. 4f). The change in global health from baseline to visit 4 demonstrated a moderate positive correlation (r = 0.48, *p* = 0.050; Fig. 4g); however, this association did not remain significant after FDR adjustment and should be considered exploratory. No meaningful correlations were observed between TTFields usage and itchy skin score at visit 4 (r = −0.07, *p* = 0.776; Fig. 4h) or with the change in itchy skin from baseline (r = 0.01, *p* = 0.979; Fig. 4i).

### Overall survival and progression-free survival

**Fig. 5:**
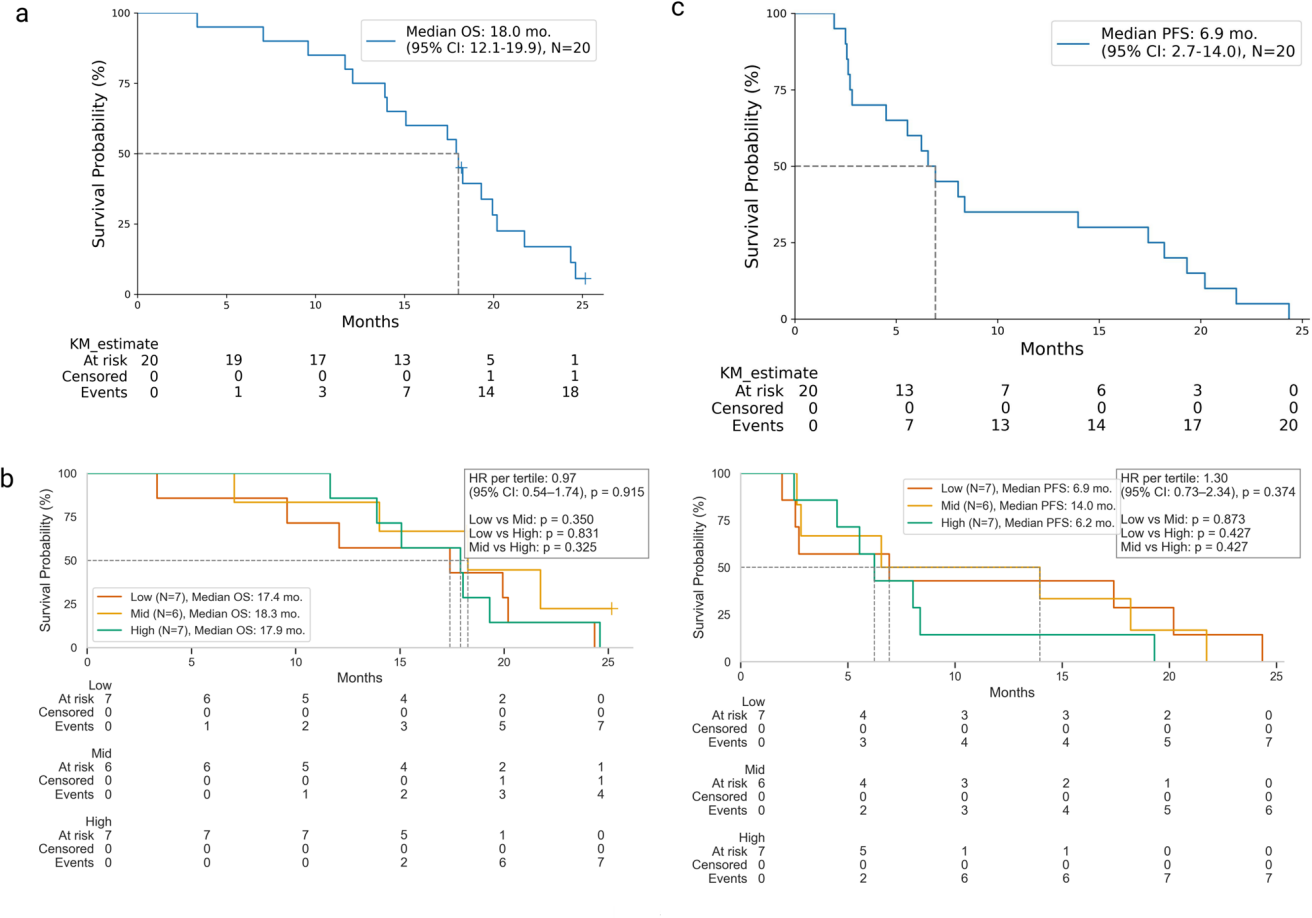
Overall Survival and Progression-Free Survival in Arm A. Kaplan–Meier estimates were calculated from the date of surgery in the exposure-conditioned Full Analysis Set of 20 patients in Arm A. Panels A and C show overall survival and progression-free survival for the full Arm A cohort, with median estimates and 95% confidence intervals. Numbers at risk, censored observations, and cumulative events are shown below the x axis. Panels B and D show exploratory overall survival and progression-free-survival curves stratified by tertiles of predicted average Tumor Treating Fields usage over the first 12 weeks. Hazard ratios and 2-sided log-rank P values are shown as plotted. These tertile analyses were exploratory and were not adjusted for multiplicity. Patient-level first-line chemotherapy regimen is shown in Fig. 1d. Regimen-stratified survival analyses were not performed because only three patients received lomustine-temozolomide

Overall survival and PFS outcomes for Arm A are shown in Fig. 5. As prespecified, OS and PFS were measured from the date of surgery. Median follow-up by reverse Kaplan–Meier was 25.2 months. Within the exposure-conditioned FAS (n = 20), median OS was 18.0 months (95% CI, 12.1–19.9). Median PFS was 6.9 months (95% CI, 2.7–14.0). The curves are shown in Fig. 5a and Fig. 5c. The wide confidence interval reflects the small sample size and limits clinical interpretation. Joint longitudinal-survival modeling and Kaplan–Meier curves stratified by usage tertiles did not identify statistically significant associations between TTFields usage and OS or PFS (Fig. 5b,d and Supplementary Fig. 8). For PFS, the hazard ratio per 1% higher daily usage was 1.02 (95% CI, 0.99–1.06, *P* = 0.21). For OS, it was 1.00 (95% CI, 0.96–1.03, *P* = 0.80). These analyses remain exploratory and underpowered, and the absence of statistically significant associations should not be interpreted as evidence of absence of a usage-survival relationship. As a prespecified sensitivity analysis, OS and PFS were also estimated in the Arm A SAF (n = 23, including three patients excluded from the FAS for insufficient compliance). In this broader population, median OS was 17.9 months (95% CI, 12.1–19.9) and median PFS was 6.6 months (95% CI, 2.7–8.4). These results are shown in Supplementary Table 4. The corresponding Kaplan–Meier curves are shown in Supplementary Fig. 9. Thus, inclusion of the three insufficient-exposure patients led to similar median OS and slightly shorter median PFS, supporting the conclusion that the primary survival estimates should be interpreted cautiously and not as proof of efficacy. Patient-level first-line treatment regimen is shown descriptively in Fig. 1d. Regimen-stratified survival analyses were not performed because only three patients received lomustine-temozolomide.

## Discussion

In this prospective multicenter phase I/II trial, initiating Tumor Treating Fields before radiotherapy and continuing them through chemoradiotherapy was feasible in patients with newly diagnosed glioblastoma. No protocol-defined TLTs occurred, device uptake was rapid, median usage until 4 weeks after radiochemotherapy exceeded the commonly cited 75% usage benchmark, and functional, cognitive, and global quality of life measures remained largely stable during the early treatment phase. The study was designed to answer a feasibility question, not to establish a biological radiosensitization effect or treatment efficacy. Together, these findings suggest that bringing Tumor Treating Fields forward into the postoperative pre-radiotherapy interval and concurrent radiotherapy phase can be operationalized, while the 0% TLT rate should be interpreted alongside the observed burden of grade 3 or higher adverse events and the narrow protocol definition of treatment-limiting toxicity [8, 15, 22, 25].

This study has several limitations. First, Arm A included only 20 evaluable patients, so the absence of observed TLTs should not be misconstrued as proof that clinically relevant toxicity has been excluded. The exact upper bound of the 95% confidence interval still permits a nontrivial event rate. Moreover, the protocol-defined treatment-limiting toxicity was narrowly focused on dermatologic endpoints and compliance thresholds, meaning that the 0% TLT rate coexisted with grade 3 or higher adverse events in 60% of patients, and a broader toxicity definition might have yielded a different feasibility conclusion. The finding of no protocol-defined TLT should therefore be read as meeting a prespecified feasibility endpoint, not as demonstrating absence of substantial toxicity overall. Second, the single-arm design precludes causal inference regarding efficacy, corticosteroid reduction, or preservation of patient-reported outcomes. Third, the exploratory survival analyses were exposure-conditioned and therefore inherently vulnerable to selection bias, because patients with insufficient compliance were excluded from the efficacy set while remaining in the safety population. Patients unable to sustain early device use may differ prognostically because of treatment tolerance, early progression, functional decline, or other unmeasured factors. To mitigate this potential bias, a sensitivity analysis including all 23 Arm A Safety Analysis Set patients was performed. Median OS (17.9 months) and PFS (6.6 months) in this broader population closely paralleled the FAS estimates (Supplementary Table 4), suggesting that exclusion of insufficient-exposure patients did not materially inflate the reported median survival estimates. Fourth, the use of two different chemotherapy regimens (Stupp protocol in 85% and CeTeG/NOA-09 in 15% of patients) introduces heterogeneity that may confound outcome interpretation, although the small number of CeTeG-treated patients limits the feasibility of stratified analyses. Fifth, diagnoses were verified by local neuropathology institutions rather than by central pathology review, which may introduce inter-institutional diagnostic heterogeneity despite use of WHO CNS5 workflows. Sixth, device-use logs were extracted by Novocure Device Support Specialists according to the protocol. Although Novocure remained blinded to clinical outcomes, did not assess endpoints, and site investigators verified data before database lock, this workflow cannot fully eliminate potential bias related to technical log transfer or completeness.

Despite these limitations, PriCoTTF has important strengths. It prospectively tested a biologically motivated treatment schedule that addresses the postoperative interval before radiotherapy, a period in which rapid early progression is common and prognostically adverse. The study also incorporated granular daily device-use data and repeated patient-centered assessments rather than reducing feasibility to toxicity alone. That design matters because a concurrent Tumor Treating Fields strategy is only clinically meaningful if patients can sustain therapeutically relevant exposure during the most demanding phase of first-line treatment [18, 19, 22].

The safety results are consistent with prior clinical experience but extend it by moving Tumor Treating Fields earlier. In the pilot prospective study by Bokstein et al., concurrent Tumor Treating Fields with radiotherapy and temozolomide was feasible and Tumor Treating Fields-related toxicity was limited predominantly to low-grade skin events [15]. In our cohort, the same overall pattern was observed despite an earlier start before radiotherapy. No protocol-defined TLTs, grade 4 dermatologic events, TLT-level moist desquamation leading to compliance loss below 50%, or permanent TTFields discontinuations for skin toxicity occurred. The one grade 3 pustular rash reviewed as an SAE occurred outside the array contact field and was not classified as TTFields + RT related by the independent safety committee. At the patient-reported outcome level, however, itchy skin worsened significantly and represented the clearest treatment-specific burden. This pattern supports feasibility and indicates that proactive dermatologic prevention, early symptom treatment, and device-use support are essential if TTFields are moved into the concurrent radiotherapy phase. This interpretation is supported by post-approval series and global surveillance datasets, which consistently show that Tumor Treating Fields-associated AEs are largely cutaneous and manageable rather than systemic [26–28].

The usage and patient-reported outcome findings are also clinically relevant. Median usage through visit 4 (i.e. 4 weeks after end of radiotherapy) was 76.5%, indicating that earlier initiation did not prevent patients from reaching the exposure range repeatedly associated with better outcomes in maintenance-phase datasets. The small sample size, restricted variability in usage, and exploratory modeling limit interpretation of the absence of a measurable usage-survival association in PriCoTTF. The transient decline in mean usage to 53.7% during radiotherapy highlights the need for proactive usage support strategies during the concurrent phase, including standardized counseling, dedicated device support visits, management of scalp symptoms, and early identification of patients at risk of disengagement. From a mechanistic perspective, the 53.7% mean usage during radiotherapy falls below the 75% threshold previously associated with improved outcomes, raising the question of whether therapeutically relevant radiosensitization can be achieved during the biologically most critical treatment window. This tension between formal feasibility and effective biological exposure warrants attention in future trial designs. Likewise, the largely stable European Organisation for Research and Treatment of Cancer and Mini-Mental State Examination/KPS trajectories, with itchy skin emerging as the main Tumor Treating Fields-specific burden, are directionally concordant with randomized and real-world experience showing preserved overall quality of life except for array-related scalp symptoms. Importantly, itchy skin was also the only domain showing significant worsening in the randomized EF-14 trial [25], reinforcing the consistency of this finding across clinical settings [22, 27].

By contrast, efficacy signals should be interpreted conservatively. Median OS of 18.0 months and PFS of 6.9 months in this exposure-conditioned cohort fall within a clinically plausible range for contemporary newly diagnosed IDH-wildtype glioblastoma populations, but cross-trial comparisons are confounded by differences in time zero, eligibility, salvage therapy, and imaging definitions of progression. Notably, the PriCoTTF Arm A cohort had a higher proportion of MGMT-unmethylated tumors (70%) compared with tested patients in the EF-14 TTFields arm (54%) [8], representing an important prognostic imbalance that must be considered when contextualizing the observed median OS of 18.0 months. Similarly, only 20% of patients had complete resections compared with 53% in EF-14 [8], further underscoring that these exploratory survival outcomes were achieved in a prognostically unfavorable population. Of note, PFS in PriCoTTF was measured from surgery, whereas EF-14 measured PFS from randomization during the maintenance phase several months later [8]. This difference in time zero must be considered when comparing PFS estimates across studies. PriCoTTF therefore does not demonstrate that concurrent initiation is superior to the established post-chemoradiotherapy approach used in EF-14. A narrower conclusion is more defensible. Earlier delivery appears sufficiently feasible to justify definitive testing of the biological hypothesis that Tumor Treating Fields may add value when introduced during the postoperative pre-radiotherapy window and during radiation, a rationale supported by preclinical radiosensitization data and by the frequency of rapid early progression before radiotherapy begins [8, 12, 29, 30].

Future research should focus on randomized estimation of efficacy, alignment of imaging end points with RANO 2.0, proactive skin-management pathways to protect usage from the start of therapy, and identification of whether earlier Tumor Treating Fields alters failure patterns or benefits selected biological or clinical subgroups. Recent observational analyses continue to suggest survival associations and possibly altered patterns of progression with Tumor Treating Fields, but these datasets cannot answer the timing question that PriCoTTF was designed to open. That question is appropriately being addressed in the ongoing EF-32/TRIDENT program [20, 31, 32].

## Conclusions

PriCoTTF provides prospective multicenter evidence that Tumor Treating Fields can be started before and during radiotherapy and can meet a prespecified feasibility endpoint based on protocol-defined treatment-limiting toxicity. The absence of protocol-defined TLTs should not be interpreted as absence of substantial toxicity overall, and whether earlier integration improves tumor control or survival remains unanswered and requires randomized evidence.

## Supplementary Information

Supplementary Material 1: Supplementary Figure 1. Schedule of Assessments Relative to Surgery and Radiotherapy. This diagram shows the timing of protocol visits relative to surgery and the radiotherapy interval. Baseline occurred 2 to 4 weeks after surgery; visit 1 occurred 1 to 2 days before radiotherapy; visit 2 occurred weekly during radiotherapy; visit 3 occurred at the end of radiotherapy with a window of plus or minus 3 days; and visit 4 occurred 4 weeks after radiotherapy with a window of plus or minus 1 week. The shaded block denotes the radiotherapy phase. Supplementary Figure 2. Simon 2-Stage Feasibility Design for Arm A. The decision tree depicts the prespecified Simon optimal 2-stage design used for Arm A. After enrollment of seven patients, the regimen would stop for lack of feasibility if 3 or more treatment-limiting toxicities occurred by 4 weeks after radiotherapy. If 2 or fewer treatment-limiting toxicities occurred, an additional 13 patients were to be enrolled. The regimen would be declared feasible if 4 or fewer of 20 patients experienced a treatment-limiting toxicity. Supplementary Figure 3. Individual Daily Tumor Treating Fields Usage Trajectories in Arm A. Each panel shows daily usage in hours for a single patient from surgery through the early post-radiotherapy period. Vertical markers indicate surgery, Tumor Treating Fields initiation, the radiotherapy interval, and visit 4. These plots illustrate patient-level heterogeneity and short interruptions; apparent post-radiotherapy increases should be interpreted in light of treatment discontinuation among some patients with low usage during radiotherapy. Supplementary Figure 4. Weekly Distribution of Tumor Treating Fields Usage in Arm A. Weekly box plots show the distribution of Tumor Treating Fields usage percentages in Arm A. Red points and the dashed red line show weekly medians. Vertical reference lines indicate radiotherapy start, radiotherapy end, and visit 4. Sample sizes per week are annotated above the boxes. Supplementary Figure 5. Individual Longitudinal Functional and Cognitive Measures. Panels A, B, and C show patient-level trajectories for Karnofsky Performance Status, dexamethasone dose, and Mini-Mental State Examination scores, respectively, across baseline, visit 3, and visit 4; overlapping trajectories may occur when patients share identical values. Dashed horizontal reference lines in the Mini-Mental State Examination panel indicate thresholds for mild and moderate cognitive impairment. Supplementary Figure 6. European Organisation for Research and Treatment of Cancer Quality of Life Questionnaire-Core 30 Domain Scores. Plots show mean scores with standard errors across baseline, visit 3, and visit 4 for core functional and symptom domains from the Quality of Life Questionnaire-Core 30. Insets display the change from baseline and the false-discovery-rate-adjusted P value for each comparison. Higher scores indicate better functioning for functional domains and greater symptom burden for symptom domains. Supplementary Figure 7. European Organisation for Research and Treatment of Cancer BN20 Domain Scores. Plots show mean scores with standard errors across baseline, visit 3, and visit 4 for brain tumor-specific symptom domains from the BN20 module. Insets display the change from baseline and the false-discovery-rate-adjusted P value for each comparison. Higher scores indicate greater symptom burden. Itchy skin was the only domain with a statistically significant worsening after multiplicity adjustment. Supplementary Figure 8. Exploratory association between predicted average TTFields usage over the first 12 weeks and survival in Arm A. Forest plot showing hazard ratios per 10% increase in predicted average usage for progression-free survival and overall survival. Supplementary Figure 9. Sensitivity analysis of overall survival and progression-free survival in Arm A. Kaplan-Meier curves compare overall survival and progression-free survival in the Arm A Full Analysis Set of 20 patients with the Arm A Safety Analysis Set of 23 patients. Both analyses use surgery as time zero. Supplementary Table 1. Sensitivity Analysis for the Estimated Treatment-Limiting Toxicity Rate. The table reports the protocol-defined treatment-limiting-toxicity rate and two sensitivity scenarios that count either the three serious adverse events or any grade 3 or higher dermatologic adverse event as a treatment-limiting toxicity. Exact confidence intervals and the exact binomial P value for the serious-adverse-event scenario are shown. Supplementary Table 2. Average Tumor Treating Fields Usage Rate Per Patient. The table lists the mean percentage of time each patient used Tumor Treating Fields from treatment initiation through visit 4.

## Data Availability

The datasets used and/or analysed during the current study are available from the corresponding author on reasonable request, subject to institutional, ethics, and data-protection restrictions.

## Abbreviations

AE: Adverse event
BMI: Body-mass index
CI: Confidence interval
CTCAE: Common Terminology Criteria for Adverse Events
EORTC: European Organisation for Research and Treatment of Cancer
FAS: Full Analysis Set
IDH: Isocitrate dehydrogenase
KPS: Karnofsky Performance Status
MGMT: O6-methylguanine-DNA methyltransferase
MMSE: Mini-Mental State Examination
OS: Overall survival
PFS: Progression-free survival
RANO: Response Assessment in Neuro-Oncology
RT: Radiotherapy
SAF: Safety Analysis Set
SAE: Serious adverse event
TLT: Treatment-limiting toxicity
TMZ: Temozolomide
TTFields: Tumor Treating Fields
