# Supplementary material for: Safety and feasibility of tumor treating fields initiated before and during radiotherapy for newly diagnosed glioblastoma: results from the Arm A feasibility cohort of a phase I/II trial (PriCoTTF)
Source: BMC Cancer. 2026 Jul 27;26:912. doi: 10.1186/s12885-026-16613-y (PMC13425971; doi:10.1186/s12885-026-16613-y)
Supplement: Supplementary file 1 — Supplementary Material 1: Supplementary Figure 1. Schedule of Assessments Relative to Surgery and Radiotherapy. This diagram shows the timing of protocol visits relative to surgery and the radiotherapy interval. Baseline occurred 2 to 4 weeks after surgery; visit 1 occurred 1 to 2 days before radiotherapy; visit 2 occurred weekly during radiotherapy; visit 3 occurred at the end of radiotherapy with a window of plus or minus 3 days; and visit 4 occurred 4 weeks after radiotherapy with a window of plus or minus 1 week. The shaded block denotes the radiotherapy phase. Supplementary Figure 2. Simon 2-Stage Feasibility Design for Arm A. The decision tree depicts the prespecified Simon optimal 2-stage design used for Arm A. After enrollment of seven patients, the regimen would stop for lack of feasibility if 3 or more treatment-limiting toxicities occurred by 4 weeks after radiotherapy. If 2 or fewer treatment-limiting toxicities occurred, an additional 13 patients were to be enrolled. The regimen would be declared feasible if 4 or fewer of 20 patients experienced a treatment-limiting toxicity. Supplementary Figure 3. Individual Daily Tumor Treating Fields Usage Trajectories in Arm A. Each panel shows daily usage in hours for a single patient from surgery through the early post-radiotherapy period. Vertical markers indicate surgery, Tumor Treating Fields initiation, the radiotherapy interval, and visit 4. These plots illustrate patient-level heterogeneity and short interruptions; apparent post-radiotherapy increases should be interpreted in light of treatment discontinuation among some patients with low usage during radiotherapy. Supplementary Figure 4. Weekly Distribution of Tumor Treating Fields Usage in Arm A. Weekly box plots show the distribution of Tumor Treating Fields usage percentages in Arm A. Red points and the dashed red line show weekly medians. Vertical reference lines indicate radiotherapy start, radiotherapy end, and visit 4. Sample sizes per week are anno [file 12885_2026_16613_MOESM1_ESM.pdf]

Supplementary Figure 1

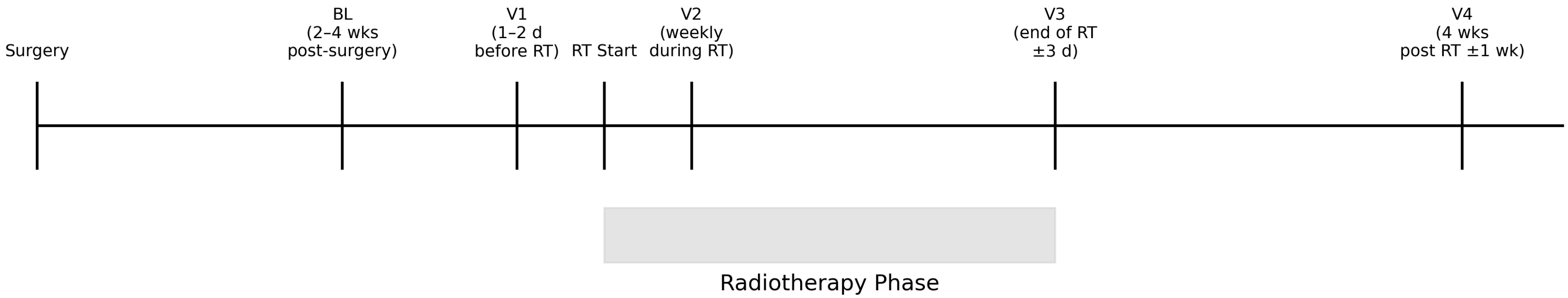

Supplementary Figure 2

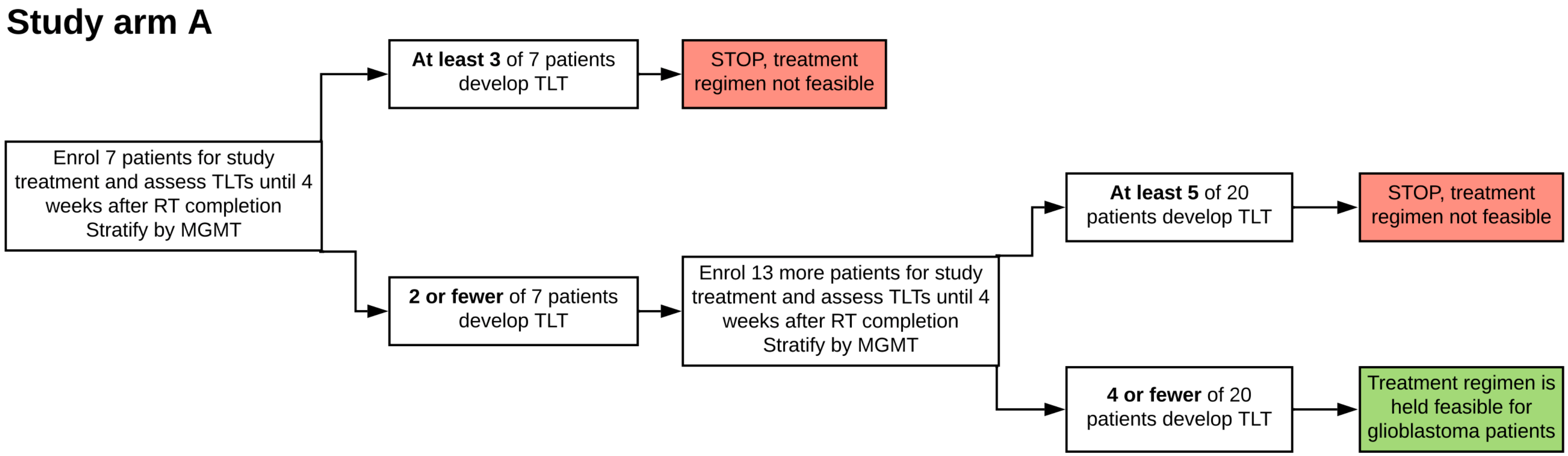

Supplementary Figure 3

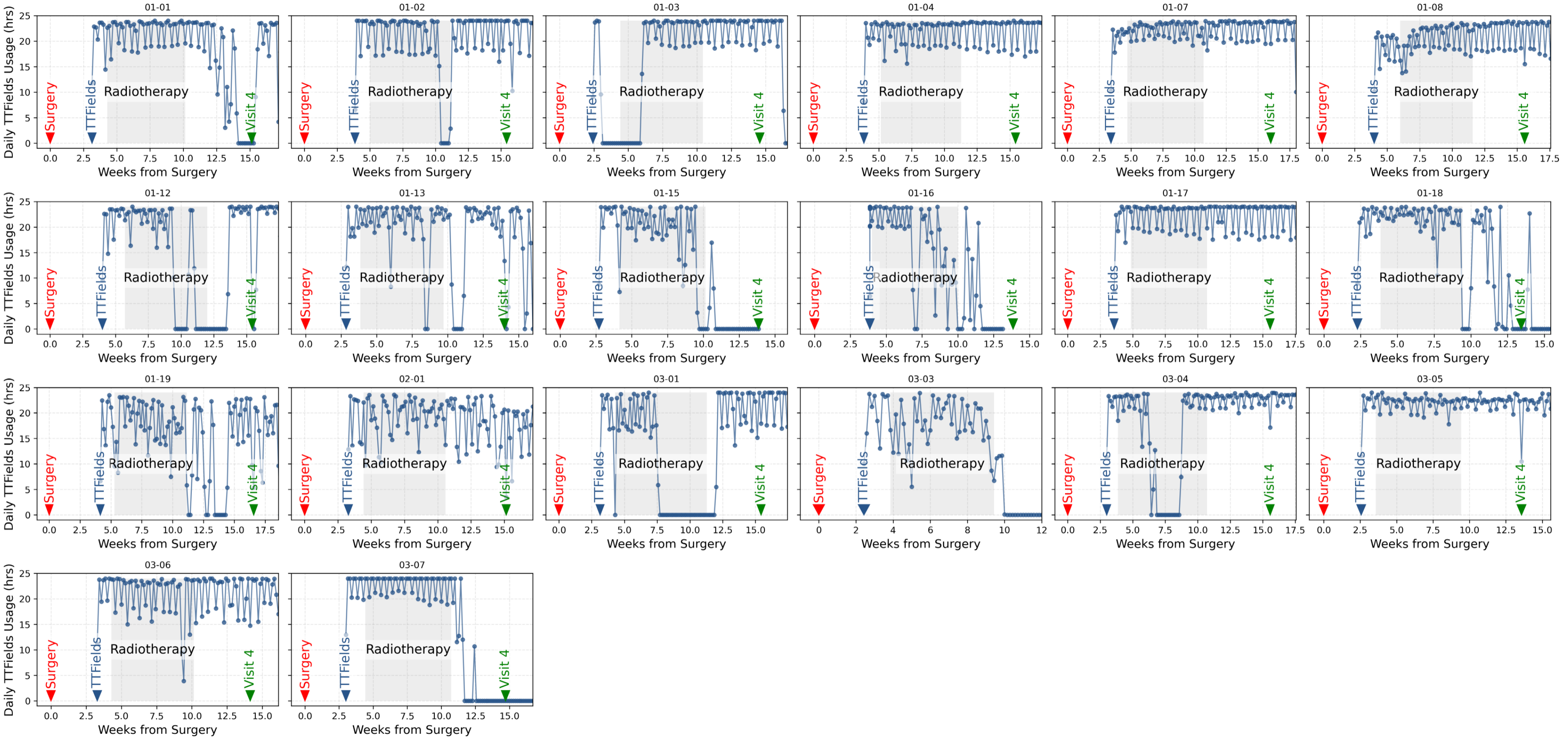

Supplementary Figure 4

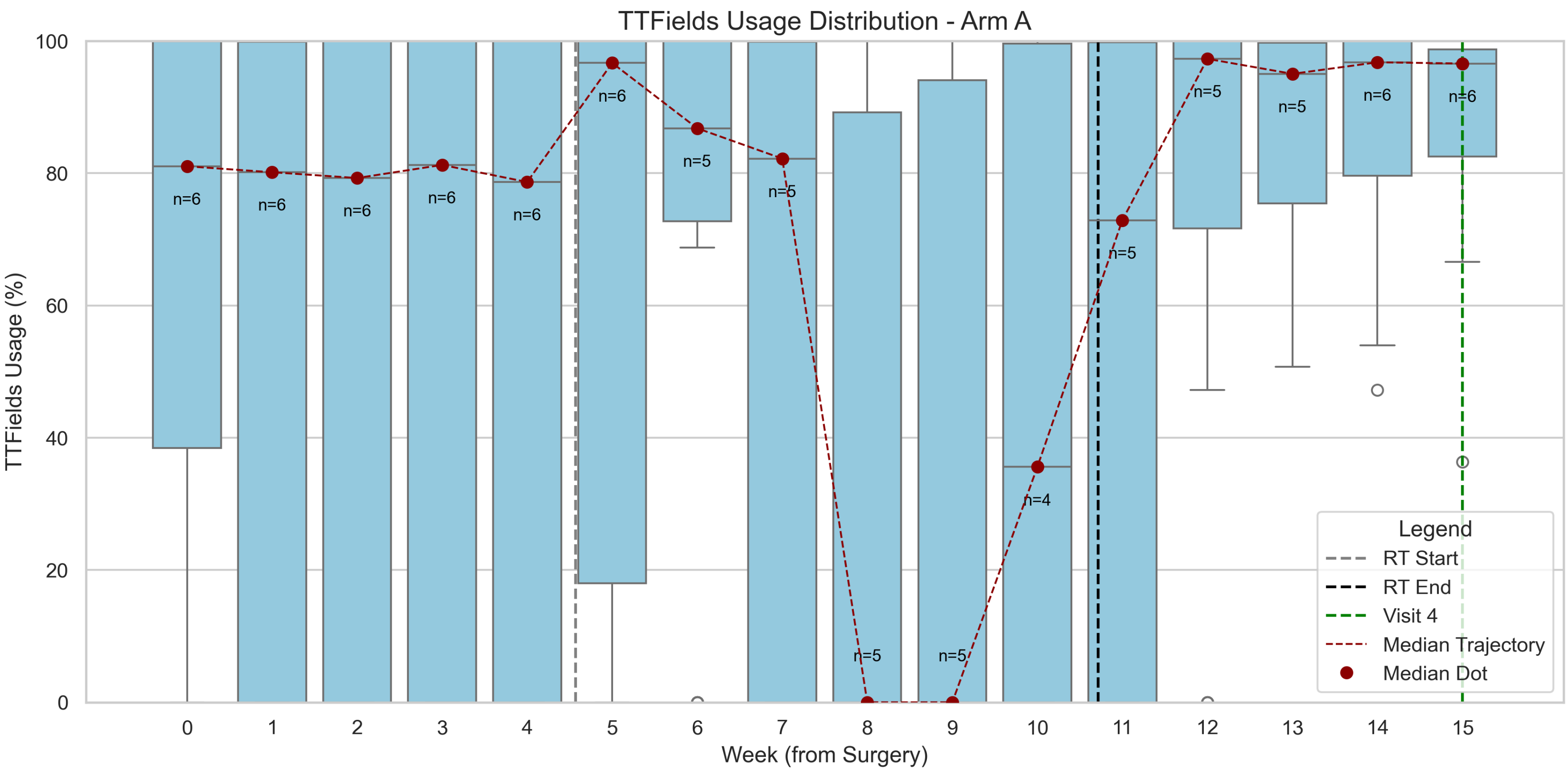

Supplementary Figure 5

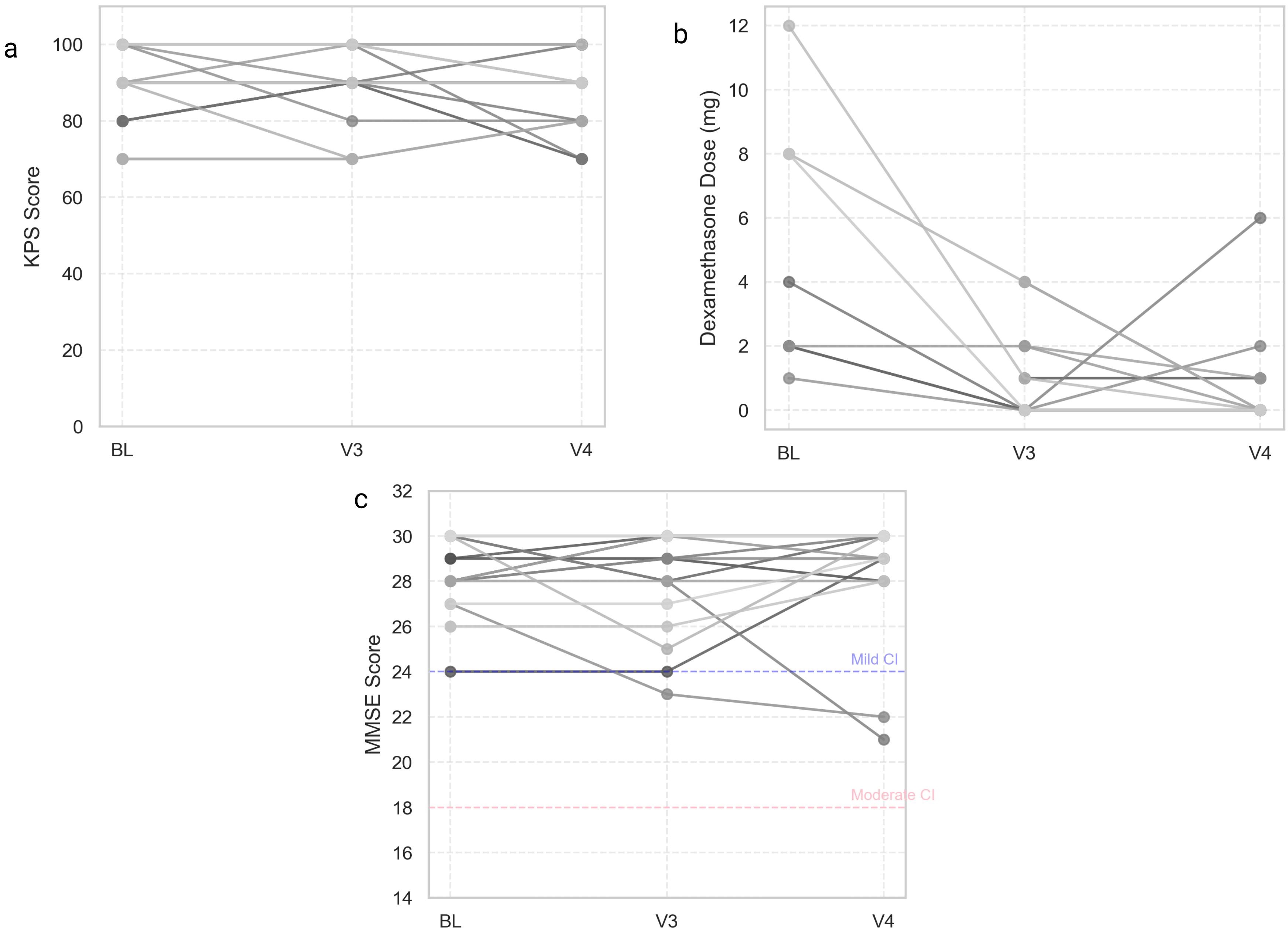

QoL C30 Domain Scores

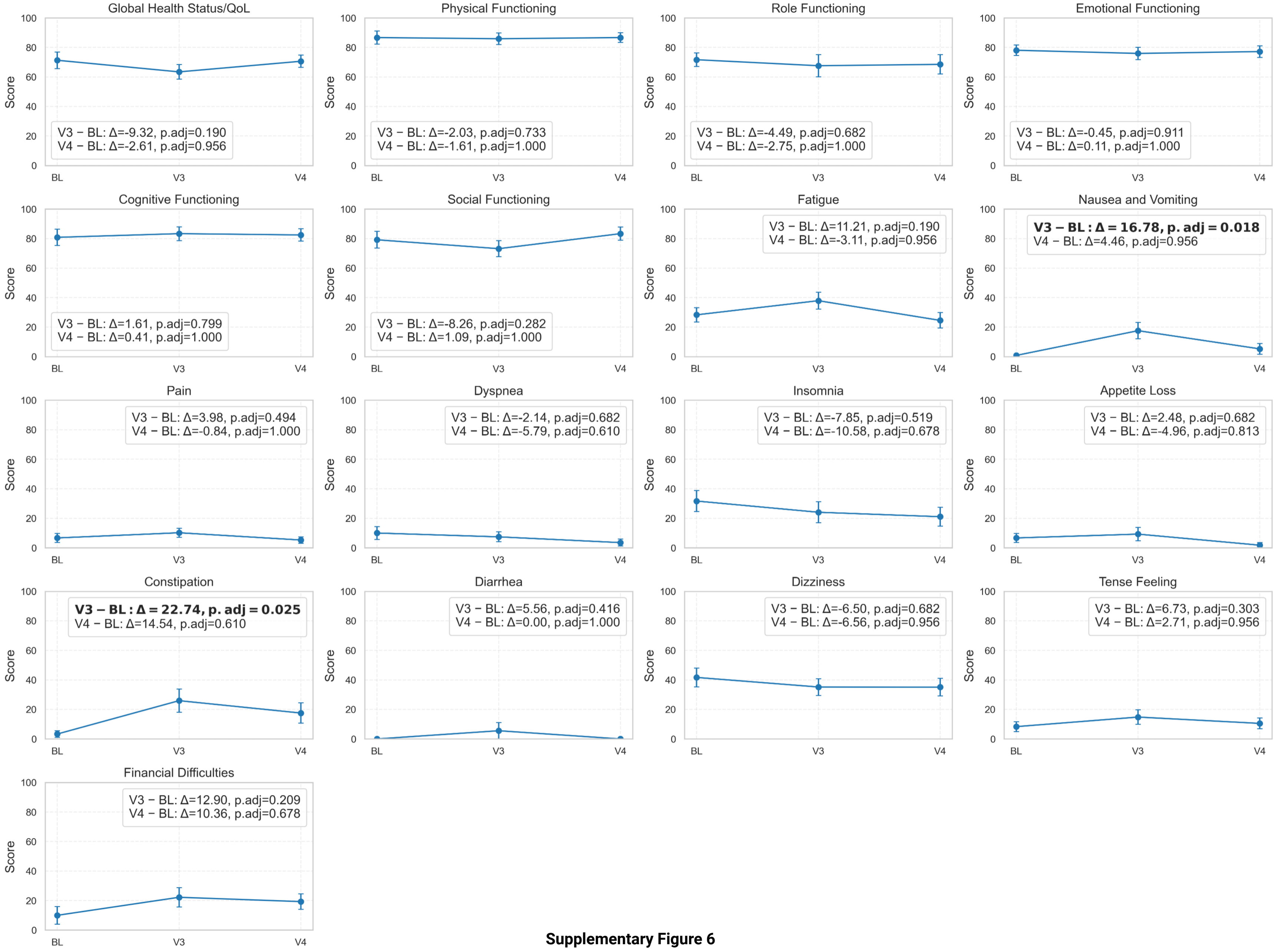

Supplementary Figure 6

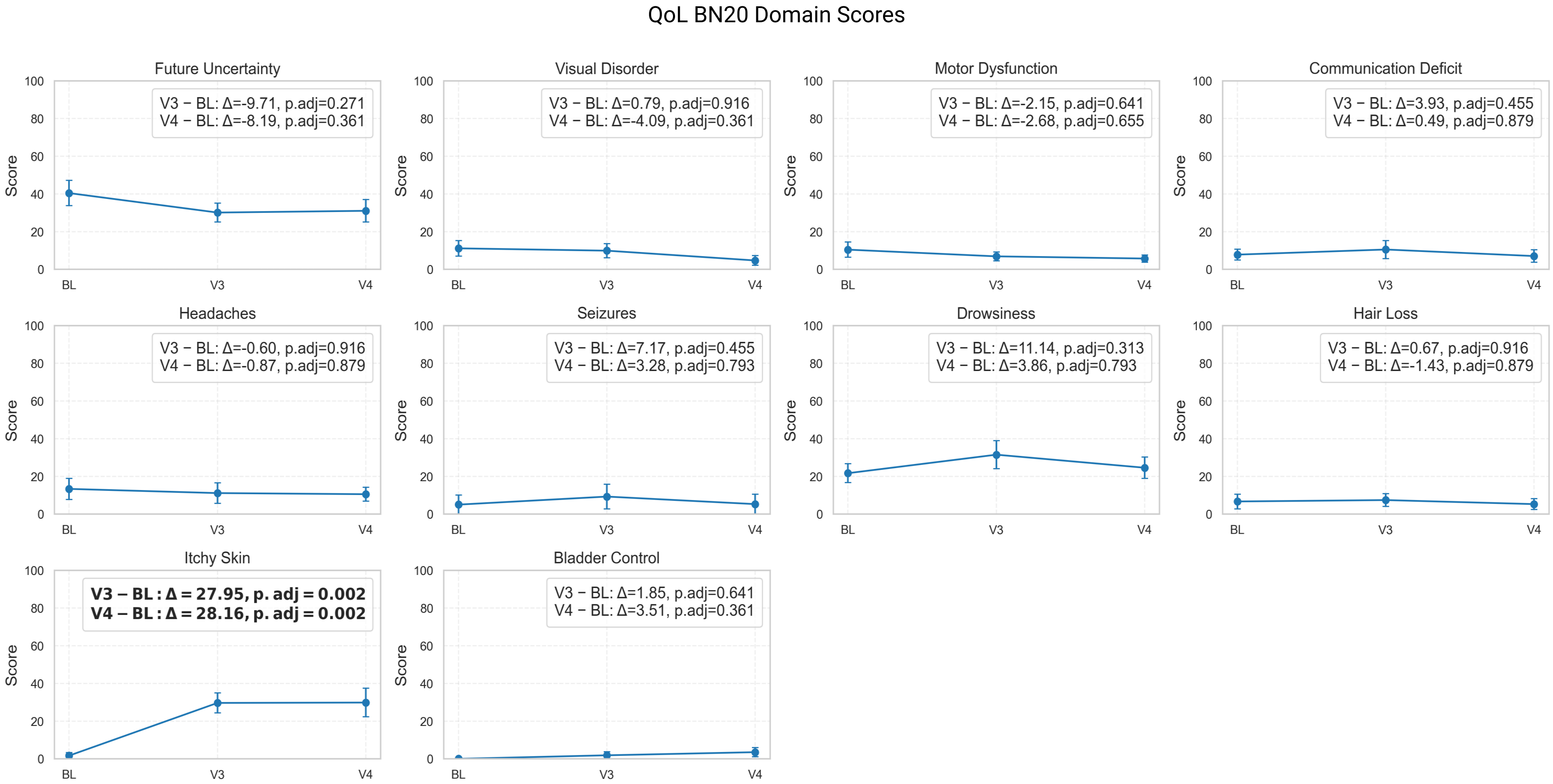

Supplementary Figure 7

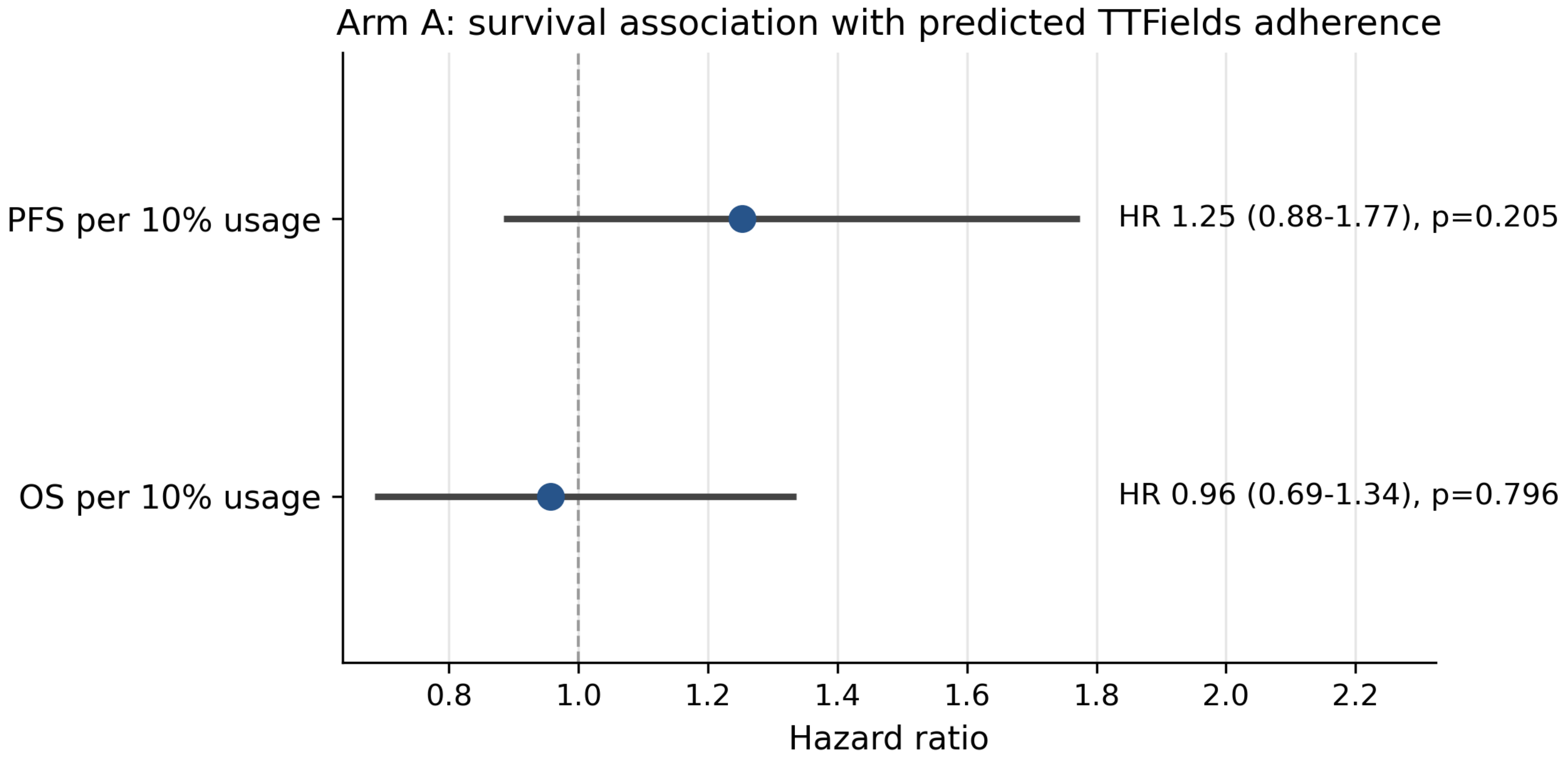
Arm A: survival association with predicted TTFields adherence
PFS per 10% usage
OS per 10% usage
HR 1.25 (0.88-1.77), p=0.205
HR 0.96 (0.69-1.34), p=0.796
0.8
1.0
1.2
1.4
1.6
1.8
2.0
2.2
Hazard ratio

Supplementary Figure 9.

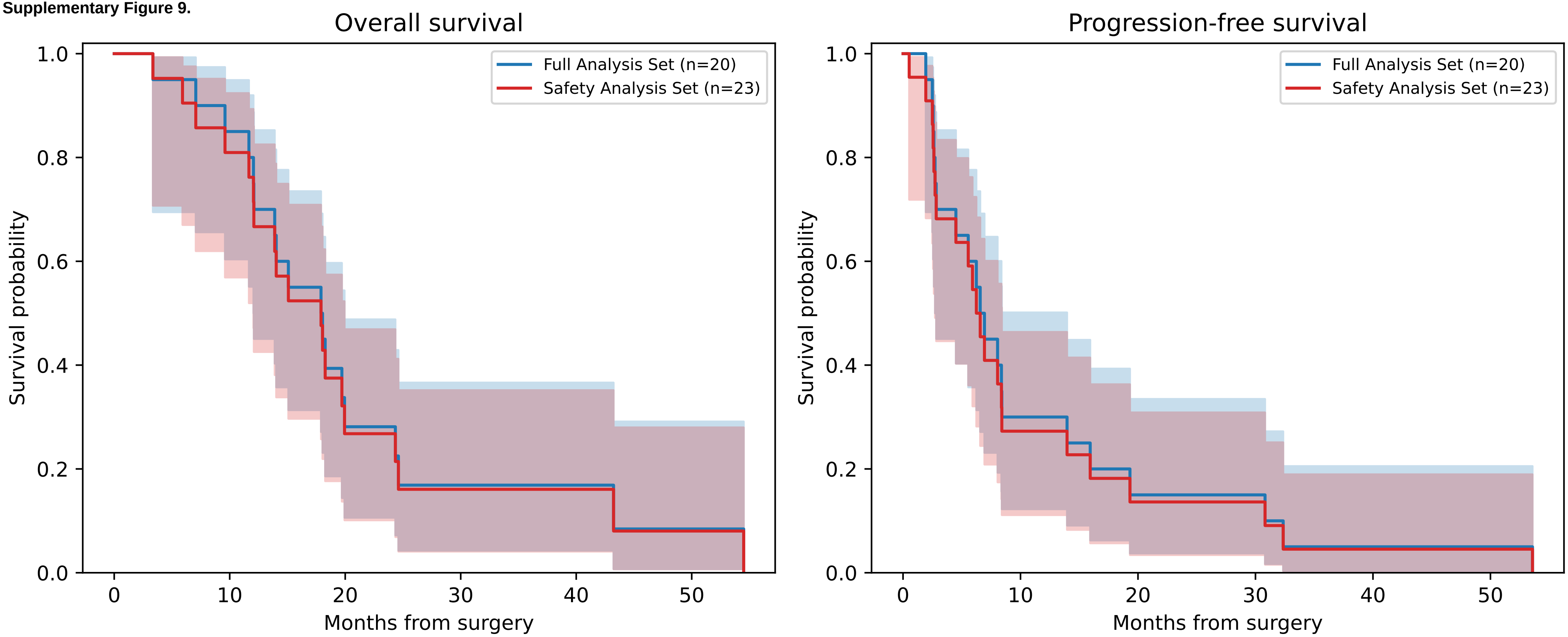

Supplementary Table 1. Sensitivity analyses using alternative treatment-limiting toxicity definitions

TLT definition	TLT, n/20	Rate	95% CI	Feasible
Protocol-defined treatment-limiting toxicity	0/20	0.000	0.000-0.168	Yes
Three serious adverse events counted as potential TLTs	3/20	0.150	0.032-0.379	Yes
Any grade 3 or higher dermatologic adverse event counted as a TLT	2/20	0.100	0.012-0.317	Yes

All sensitivity definitions remained below the prespecified 40 percent non-feasibility threshold. The one-sided exact binomial P value for the serious-adverse-event scenario versus a 40 percent threshold was 0.016. Confidence intervals are exact Clopper-Pearson intervals.

Supplementary Table 2. Average Tumor Treating Fields usage rate per patient (Arm A Full Analysis Set, n=20).

Patient ID	Usage rate (%)
01-01	77
01-02	84
01-03	70
01-04	90
01-07	92
01-08	85
01-12	61
01-13	80
01-15	53
01-16	57
01-17	92
01-18	58
01-19	64
02-01	80
03-01	53
03-03	51
03-04	76
03-05	90
03-06	87
03-07	68

Usage rate is the mean percentage of time each patient used Tumor Treating Fields from treatment initiation through visit 4. Median across the 20 patients was 76.5%.

Supplementary Table 3. Association between predicted 12-week TTFields usage and survival in the Arm A Full Analysis Set

Endpoint	n (events)	HR per 1% higher predicted usage	95% CI	P value	Concordance	Partial AIC
PFS	20 (20)	1.02	0.99-1.06	0.21	0.51	85.09
OS	20 (18)	1.00	0.96-1.03	0.80	0.55	82.21

Hazard ratios are reported per one percentage point higher predicted TTFields usage. The Arm A Full Analysis Set included 20 patients. The analyses were exploratory and descriptive.

Supplementary Table 4. Sensitivity analysis of overall survival and progression-free survival in Arm A

Set	n	OS events	OS censored	Median OS, months	OS 95% CI	PFS events	PFS censored	Median PFS, months	PFS 95% CI
FAS	20	18	2	18.0	12.1-19.9	20	0	6.9	2.7-14.0
SAF	23	19	4	17.9	12.1-19.9	22	1	6.6	2.7-8.4

Overall survival and progression-free survival were measured from surgery. The Full Analysis Set included 20 patients with sufficient early TTFields exposure. The Safety Analysis Set included all 23 Arm A patients who started TTFields and radiotherapy.

Supplementary Table 5. Dermatologic adverse events of any grade in the Arm A Full Analysis Set (n=20).

Preferred term	Grade 1	Grade 2	Grade 3	Total events	Patients
Administration site dermatitis	5	3	0	8	5
Application site rash	1	0	0	1	1
Dermatitis	1	0	0	1	1
Dermatitis allergic	1	0	0	1	1
Drug reaction with eosinophilia and systemic symptoms	0	0	1	1	1
Erythema	3	2	0	5	3
Pruritus	6	0	0	6	5
Rash	3	3	1	7	2
Rash pustular	0	0	1	1	1
Skin erosion	1	0	0	1	1
Skin injury	2	0	0	2	2
Skin irritation	3	0	0	3	2
Skin ulcer	1	1	0	2	1

Any-grade dermatologic adverse events occurred in 18 of 20 patients (90%) and were predominantly grade 1-2. Grade 3 or higher dermatologic events occurred in 2 of 20 patients (10%): one grade 3 pustular rash, one grade 3 rash, and one grade 3 drug reaction with eosinophilia and systemic symptoms. No grade 4 dermatologic event and no dermatologic treatment-limiting toxicity occurred. Counts are events; Patients gives the number of distinct patients with at least one event of that term.
